# Oral Bioavailability Enhancement of Melanin Concentrating Hormone, Development and In Vitro Pharmaceutical Assessment of Novel Delivery Systems

**DOI:** 10.3390/pharmaceutics14010009

**Published:** 2021-12-21

**Authors:** Dóra Kósa, Ágota Pető, Ferenc Fenyvesi, Judit Váradi, Miklós Vecsernyés, István Budai, József Németh, Pálma Fehér, Ildikó Bácskay, Zoltán Ujhelyi

**Affiliations:** 1 Department of Pharmaceutical Technology, Faculty of Pharmacy, University of Debrecen, Nagyerdei Körút 98, 4032 Debrecen, Hungary; kosa.dora@pharm.unideb.hu (D.K.); peto.agota@pharm.unideb.hu (Á.P.); fenyvesi.ferenc@pharm.unideb.hu (F.F.); varadi.judit@pharm.unideb.hu (J.V.); vecsernyes.miklos@pharm.unideb.hu (M.V.); feher.palma@pharm.unideb.hu (P.F.); ildiko.bacskay@pharm.unideb.hu (I.B.); 2 Doctoral School of Pharmaceutical Sciences, University of Debrecen, Nagyerdei Körút 98, 4032 Debrecen, Hungary; 3 Institute of Healthcare Industry, University of Debrecen, Nagyerdei Körút 98, 4032 Debrecen, Hungary; 4 Faculty of Engineering, University of Debrecen, Ótemető Utca 2-4, 4028 Debrecen, Hungary; budai.istvan@eng.unideb.hu; 5 Department of Pharmacology and Pharmacotherapy, University of Debrecen, Nagyerdei Körút 98, 4032 Debrecen, Hungary; nemeth.jozsef@med.unideb.hu

**Keywords:** peptide carriers, bioavailability, alginate beads, penetration enhancers, biocompatibility investigation, MTT test, Caco-2 cells

## Abstract

The rapid progress in biotechnology over the past few decades has accelerated the large-scale production of therapeutic peptides and proteins, making them available in medical practice. However, injections are the most common method of administration; these procedures might lead to inconvenience. Non-invasive medications, such as oral administration of bio-compounds, can reduce or eliminate pain and increase safety. The aim of this project was to develop and characterize novel melanin concentrating hormone (MCH) formulations for oral administration. As a drug delivery system, penetration enhancer combined alginate beads were formulated and characterized. The combination of alginate carriers with amphiphilic surfactants has not been described yet. Due to biosafety having high priority in the case of novel pharmaceutical formulations, the biocompatibility of selected auxiliary materials and their combinations was evaluated using different in vitro methods. Excipients were selected according to the performed toxicity measurements. Besides the cell viability tests, physical properties and complex bioavailability assessments were performed as well. Our results suggest that alginate beads are able to protect melanin concentrating hormones. It has been also demonstrated that penetration enhancer combined alginate beads might play a key role in bioavailability improvement. These formulations were found to be promising tools for oral peptide delivery. Applied excipients and the performed delivery systems are safe and highly tolerable; thus, they can improve patients’ experience and promote adherence.

## 1. Introduction

Endogenous peptides and proteins are the building blocks of life, as they play an important role in the regulation of different life processes by forming a multitude of hormones, enzymes and antibodies [1]. The rapid progress in biotechnology over the past few decades has accelerated the large-scale production of therapeutic peptides and proteins, making them available in medical practice [2,3]. Despite inoculated peptides having saved millions of lives, the importance of alternative routes of peptide administration is not questionable [3]. The development of oral protein delivery systems has been a constant challenge to pharmaceutical technology, as it requires overcoming several obstacles resulting from peptides’ unfavorable properties [4]. Frequent peptide degradation in the GI tract, low epithelial permeability and amphiphilicity all block the way to the success of oral pharmaceutical formulations [5]. Despite the low patient compliance due to many factors such as needle phobia, at this point, injections are still the standard procedure of protein and peptide administration due to their poor oral bioavailability [6]. Despite the fact that recently many peptides, such as semaglutide, octreotide, and salmon calcitonin, have been tested for oral administration in phase III trials, only semaglutide have completed a trial successfully [7]. An oral GLP-1 receptor agonist semaglutide (Rybelsus^®^) was approved by the FDA in 2019, representing the first orally available medicine in the treatment of type 2 diabetes [8].

The encapsulation of peptides into finely divided bioadhesive polymer carrier systems is a preferable and widespread method to improve oral bioavailability of peptide drugs due to their beneficial properties [9,10]. Natural polymers are biosafe and highly inert carrier matrices as they not only protect the drug from gastrointestinal degradation but also improve absorption [11,12,13]. Moreover, polymeric small particles improve the effectiveness of proteins by providing controlled drug release [14,15]. Alginate particles showed potential results as carriers designed for oral administration of proteins and peptides [16,17,18,19,20]. Alginate is a biocompatible and biodegradable natural polysaccharide having excellent biocompatibility and mucoadhesive biodegradability. In the presence of divalent cations, such as calcium, alginate forms insoluble ionic cross-linked complexes while incorporating the drug [18,21,22,23]. Adding different permeation enhancers to the delivery system can be also an applicable strategy as they may facilitate and even enhance oral absorption. Many studies proved that lipid-based surface active agents are vital tools for the oral delivery of peptides due to the observation that they are able to modulate transcellular and paracellular pathways [2,24,25,26]. It has been demonstrated that using lipid excipients in these formulations is advantageous due to their multiplicity, safety and adaptability [27]. The combination of these promising tools had not been investigated particularly.

The aim of this research was to develop innovative solid oral delivery systems for peptide delivery with improved oral bioavailability. As a model peptide, a well-defined molecule was selected. Melanin concentrating hormone (MCH) plays a crucial role in the regulation of nutrition behavior, energy homeostasis and food intake in all living beings [28,29,30,31]. Moreover, the importance of MCH had been demonstrated not only in central nervous system regulation but also in the periphery [32,33,34]. In our previous studies we performed several experiments with this natural cyclic nonadecapeptide. These experiments ensure valuable assessment of our results by the developed radioanalytical (RIA) method [35]. Peptide-loaded calcium cross-linked alginate beads were formulated by a controlled gelification method in order to protect the model peptide [36]. Characterization of drug release from the beads was performed by an in vitro dissolution test. Since there are no available data regarding the enzymatic stability and intestinal permeability of MCH, we also evaluated enzymatic degradation and permeability of the peptide and peptide-loaded microbeads as well. Moreover, we implemented our formulation study objectives by lipid-based permeation enhancers application. Several permeation enhancers were selected and incorporated into the beads in order to increase intestinal absorption. The role of these compounds has already been tested and described [37,38]. Compositions were examined and selected according their physical properties and stability. Since safety is an indispensable aspect of pharmaceutical formulations, biological properties of the excipients and blends was also evaluated. To determine the interaction between the formulated dosage forms and in vitro human tissue, a Caco-2 immortalized cell line was deployed. The Caco-2 adenocarcinoma cell line is a reliable in vitro model of human intestinal processes [39]. The biocompatibility of these compounds has been investigated by cell viability assays. To complete the assessment, its physical characteristics, such as morphology, size distribution and swelling behavior, were also determined in each case.

## 2. Materials and Methods

### 2.1. Materials

Melanin concentrating hormone was synthetized by Gábor Tóth (Department of Medical Chemistry, Faculty of Medicine, University of Szeged). Low viscosity grade sodium alginate was obtained from BÜCHI Labortechnik AG (Flawil, Switzerland). Calcium chloride dihydrate was purchased from VWR International (Debrecen, Hungary). Labrasol (Caprylocaproyl Prolyoxyl-8-glycerides) and Transcutol HP (Diethylene glycol monoethyl ether), were obtained from Gattefossé (Saint-Priest, France). Pepsin from porcine gastric mucosa (≥400 unit/mg protein) and pancreatin from porcine pancreas (≥3xUSP specifications) were purchased from Sigma-Aldrich (St. Louis, MI, USA). The human adenocarcinoma cancer cell line (Caco-2) originated from the European Collection of Cell Cultures (ECACC, Public Health England, Salisbury, UK). MTT reagent 3-(4,5-Dimethylthiazol-2-yl)-2,5-diphenyltetrazolium bromide, and buffer solutions such as Hank’s Balanced Salt Solution (HBSS) and phosphate buffered saline (PBS), were purchased from Sigma-Aldrich (St. Louis, MI, USA). Cell maintenance solutions, such as Dulbecco’s Modified Eagle’s Medium (DMEM), heat-inactivated fetal bovine serum (FBS), L-glutamine, a non-essential amino acids solution, and a penicillin-streptomycin solution, were ordered from Sigma-Aldrich (St. Louis, MI, USA). TrypLE™ Express Enzyme (no phenol red) was bought from Thermo Fisher Scientific (Waltham, MA, USA). Ninety six-well cell plates, twenty four-well cell plates and culturing flasks were obtained from VWR International (Debrecen, Hungary). ThinCert™ 24-well cell culture inserts were supplied by Greiner Bio-One Hungary Kft. (Mosonmagyaróvár, Hungary).

### 2.2. Methods

#### 2.2.1. Preparation of Sodium-Alginate and CaCl_2_ Solutions

A total of 1.50% (*w*/*v*%) polymer solution was prepared by dissolution of 3.30 g of low viscosity grade sodium-alginate in 200 mL distilled water. The solution was mixed for 180 min at room temperature (25 °C) under vigorous stirring (300 rpm) to obtain a homogeneous solution. For the CaCl_2_ solution (100 mM), 14.70 g calcium chloride dihydrate was dissolved in 1000 mL deionized water.

#### 2.2.2. Preparation of Peptide-Loaded Alginate Beads

The MCH-loaded alginate microbeads were prepared by a controlled polymerization method using a Büchi Encapsulator B-395 Pro apparatus. The peptide was finely distributed in 40 mL of 1.50% sodium-alginate solution combined with the 0.01 *v*/*v*% of penetration enhancers (Labrasol, Transcutol HP). Mixtures were loaded into a 40 mL BD LuerLock type syringe. The polymer–pharmacon mixture was forced into the pulsation chamber by a syringe pump at speed 5.00 mL/min. The solution was passed through an electrical field between the nozzle (80 µm, 200 µm, 1000 µm). A 1000 V set electrode separated the alginate solution into equal size droplets by 1800 Hz frequency. The alginate beads were left to harden for 15 min in calcium-chloride solution. The finely divided particles were washed with hardening solution and filtered on a 0.4 µm pore size membrane by a vacuum pump and freeze dried for 24 h at −110 °C [36].

#### 2.2.3. Encapsulation Efficiency

To determine the encapsulated drug content in the beads, a 1 mL sample was measured from 100 mM of the calcium-chloride hardening solution right after formulation. Drug concentration was determined by radioimmunoassay (RIA) [35]. Encapsulation efficiency (EE) was calculated by the following equation:

(1) $$EE=\left(\frac{TQ-HQ}{TQ}\right)\times 100$$

where *HQ* is the drug quantity present in the hardening solution, and *TQ* is the theoretical drug content in the beads [21]. In each case experiments were performed in triplicate.

#### 2.2.4. Study of Swelling Behavior

The swelling behavior of the alginate spheres was determined gravimetrically. A total of 85 mg dry beads were weighed and soaked in 100 mL distilled water at room temperature. The beads were removed after 1 h, and the excess wetness was eliminated with vacuum filtration. The equilibrium water uptake was calculated using the following equation:

(2) $$EWU=\left(\frac{Ws-Wd}{Ws}\right)\times 100$$

where *Ws* is the weight of swollen beads and *Wd* is the initial weight of the dry beads [40].

#### 2.2.5. Scanning Electron Microscopy

Electron microscopic analysis of the shape, size and surface area of the particles was performed using a Hitachi Tabletop microscope (TM3030 Plus) (Hitachi High-Technologies Corporation, Tokyo, Japan). Samples were attached to a plate covered with double-sided adhesive tape. An accelerating voltage of 5 kV was used during micrography.

#### 2.2.6. Particle Size Distribution

Particle size distribution of the beads formulated with a 200 μm nozzle was performed with a HoribaPartica LA-950V2 (Horiba, Ltd., Kyoto, Japan) laser diffraction particle size analyzer, operated in wet mode with distilled water (1000× dilution), performing at least three parallel measurements on each sample.

#### 2.2.7. In Vitro Dissolution

The rotating paddle method by Erweka DT 800 apparatus (Erweka GmbH, Langen, Germany) was applied to characterize drug release from the particles. In vitro dissolution was evaluated as 400 mg of the formulated dry beads were placed in 100 mL buffer phosphate solution (pH = 6.80) at 37 °C. A total of 1000 µL of the dissolution media was collected at determined time intervals (0., 5., 15., 30., 60., 90., 120. min). The drug concentration in each sample was analyzed by radioimmunoassay [35].

#### 2.2.8. Enzymatic Stability

Enzymatic degradation in the presence of proetolytic enzymes pepsin and pancreatin was investigated [41]. Peptide-loaded particles were added to 100 mL of artificial gastric fluid (simulated gastric fluid—SGF) with pepsin, or artificial intestinal fluid (simulated intestinal fluid—SIF) with pancreatin and incubated at 37 °C under moderate stirring at 100 rpm. SGF and SIF were prepared as per European Pharmacopoeia specifications. A total of 1000 µL samples were collected at determined time intervals for 120 min, and an equal volume of ice-cold reagent was added to stop the enzymatic reaction (0.10 M NaOH for SGF and 0.10 M HCl for SIF). Samples were analyzed by radioimmunoassay [35].

#### 2.2.9. Cell Culturing

A Caco-2 immortalized human adenocarcinoma cell line was purchased from The European Collection of Cell Cultures (ECACC). Cells were seeded and maintained in plastic cell culture flasks in Dulbecco’s Modified Eagel’s medium (DMEM Sigma-Aldrich Ltd., Budapest, Hungary). Cell culturing media was supplemented with 2 mM L-glutamine, 100 mg/L gentamycin (Sigma-Aldrich Ltd., Budapest, Hungary) and 10% heat inactivated fetal bovine serum (FBS) (Sigma-Aldrich Ltd., Budapest, Hungary). Cells were stored in incubators at 37 °C in a 5% CO_2_ atmosphere. The colon epithelial Caco-2 cell line forms a single cell layer with well-defined tight junctions.

#### 2.2.10. Cell Viability Assay

Caco-2 cell viability was evaluated using the MTT method. Cells were harvested and seeded at a density of 10^4^ cells/well on flat-bottom 96-well tissue culture plates. Cells were allowed to grow for 7 days under the abovementioned conditions. For the viability measurements, the DMEM was removed. Cells were incubated with the applied penetration enhancer (Labrasol, Transcutol HP; Gattefossé, Saint-Priest, France) solutions, all other components and blends for 30 min. Samples were removed and cells were washed with buffer solution. Mitochondrial activity of the cells was determined after 3 h incubation with MTT at the concentration of 0.50 mg/mL. Dark blue formazan crystals were dissolved in acidic isopropanol (isopropanol:1.00 N hydrochloric acid = 25:1). The absorbance of dissolved formazan crystals correlates with the ratio of the living cells. Absorbance was measured with a FLUOstar OPTIMA Micro-plate Reader (BMG LABTECH, Offenburg, Germany) at 570 nm against a 690 nm reference. Cell viability was demonstrated as the percentage of the untreated control.

#### 2.2.11. Permeability Test

A human colon adenocarcinoma cell line was used as the model of human intestinal absorption of MCH. Caco-2 cells were seeded on 12-well ThinCert™ transwell polyester inserts (pore size: 0.40 µm; Greiner Bio-One International, Mosonmagyaróvár, Hungary) at a density of 2 × 10^5^ cells/cm^2^ and grown for 21–28 days. Transepithelial electrical resistance (TEER) was measured every 2 days. Measurements were begun when TEER values of the inserts reached 1000 Ω×cm^2^ value. The culture medium in the apical and basolateral compartments was replaced with HBSS and the monolayers were pre-incubated for 30 min at 37 °C. After that, the experimental solution was pipetted to the apical chambers. In transport experiments, we have studied the permeability of different types of samples; one containing only peptide, another with the same amount of MCH containing 0.01% Labrasol and/or 0.01% Transcutol HP as a penetration enhancer. A total of 300 mg of the samples were dissolved in 60 mL of PBS buffer for 60 min. As a negative control HBBS was examined. The permeated amount of MCH was determined after 60 min. These samples were collected from the basolateral compartment and replaced with fresh HBSS. Samples were analyzed by radioimmunoassay. To complete the investigation, membrane function was monitored by transepithelial electrical resistance measurements.

#### 2.2.12. Transepithelial Electrical Resistance Measurements

Transepithelial electrical resistance was measured with Millipore Millicell-ERS 00001 equipment (Merck, Waltham, MA, USA). During the transmembrane experiments, TEER values were determined every 15 min. As a follow-up, measurements were continued in the following 24 h to investigate the recovery.

#### 2.2.13. Statistical Analysis

Data were handled and analyzed using Microsoft Excel 2013 and SigmaStat 4.0 (version 3.1; SPSS, Chicago, IL, USA, 2015), and herein presented as means ± SD. Comparisons of results of the in vitro dissolution test, enzymatic stability assessment, permeability test and MTT cell viability assays were performed with one-way ANOVA and repeated-measures ANOVA followed by Tukey or Dunnett post testing. Difference of means was regarded as significant in the case of *p* < 0.05. All experiments were carried out in quintuplicates and repeated at least five times.

## 3. Results

### 3.1. Formulated MCH Beads

MCH encapsulated in different alginate formulations. Selected compositions described at Table 1.

### 3.2. Encapsulation Efficiency

**Figure 1:**
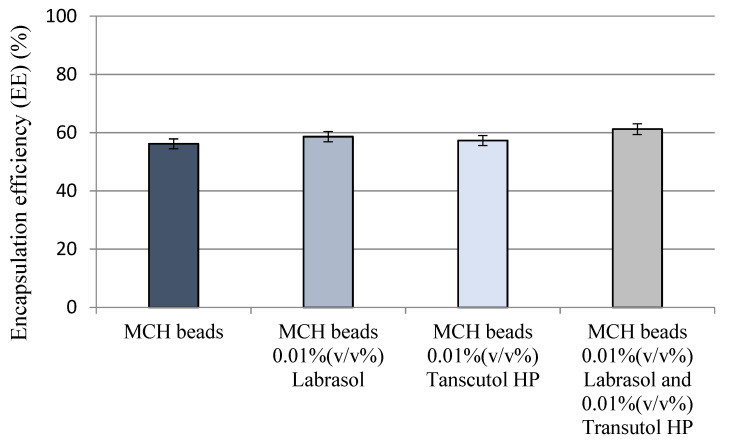
Results of encapsulation efficiency measurements regarding the different formulations containing MCH. Although there is no major difference in encapsulation efficiency between the formulations, beads containing both Labrasol and Transcutol HP as penetration enhancers showed the highest EE value. Each data point represents the mean ± SD, *n* = 3.

Encapsulation efficiency measurements resulted in at least 56%. The lowest EE was calculated in the case of simple MCH beads. Labrasol and Transcutol HP supplemented beads showed the highest EE although no significant differences had been evaluated between the closing ability of the compositions. Results of encapsulation efficiency measurements are presented in Figure 1.

### 3.3. Study of Swelling Behavior

**Figure 2:**
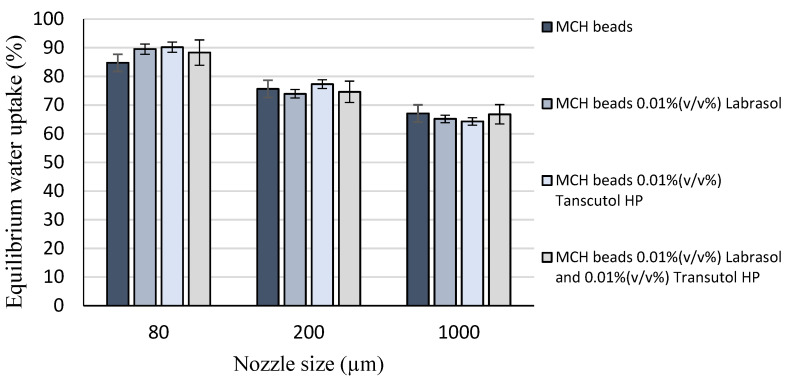
Bead swelling behavior according to the nozzle size applied for the formulation. Equilibrium water uptake is highly affected by particle size. Each data point represents the mean ± SD, *n* = 5.

Bead swelling behavior investigation results are presented in Figure 2. It is shown that the value of equilibrium water uptake depends on the particle size of beads, as the smaller is the diameter and thus the larger is the specific surface area, the higher is the water uptake capacity. These data might be useful during the formulation since swelling behavior influences the applicability.

### 3.4. Scanning Electron Microscopy

**Figure 3:**
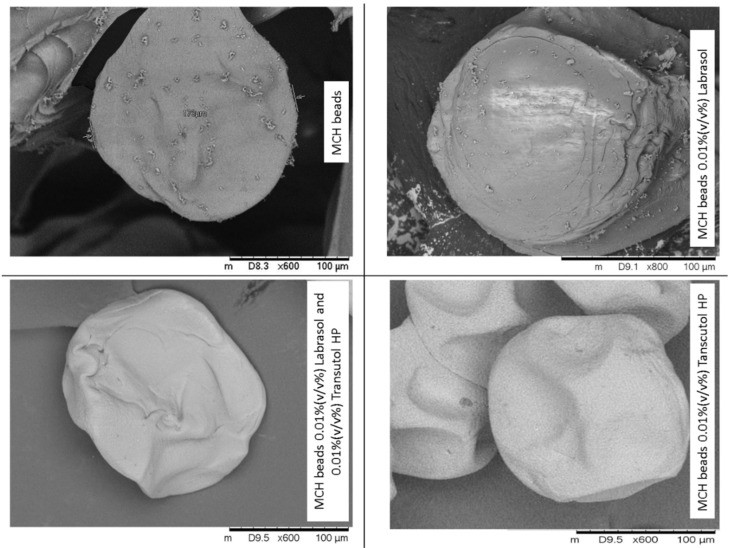
Scanning electron microscopy images of the different formulated sodium-alginate beads containing MCH. During the drying process some squashes occurred on the bead surface.

Scanning electron microscopy images of the dry MCH-loaded alginate beads are shown in Figure 3. The surface morphology of the represented microspheres showed spherical shaped beads having some squashes on the surface, probably due to the drying process. The SEM observation also demonstrated that the diameter of microbeads is consistent with the results of particle size distribution.

### 3.5. Particle Size Distribution

**Figure 4:**
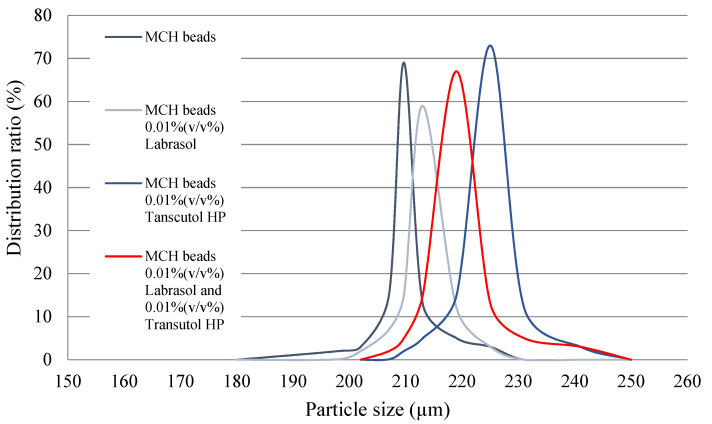
Results of laser diffraction particle size distribution according to the volume moment mean values. Particle size of the beads is close to the theoretical 200 µm regarding all four formulations.

Microbeads were analyzed by a HoribaPartica LA-950V2 laser diffraction particle size analyzer. The results of particle size distribution are reported in Table 2 with the relative standard deviation value. According to the volume moment mean values (Figure 4), the particle size of the formulated microbeads is close to the theoretical 200 µm.

### 3.6. In Vitro Dissolution

**Figure 5:**
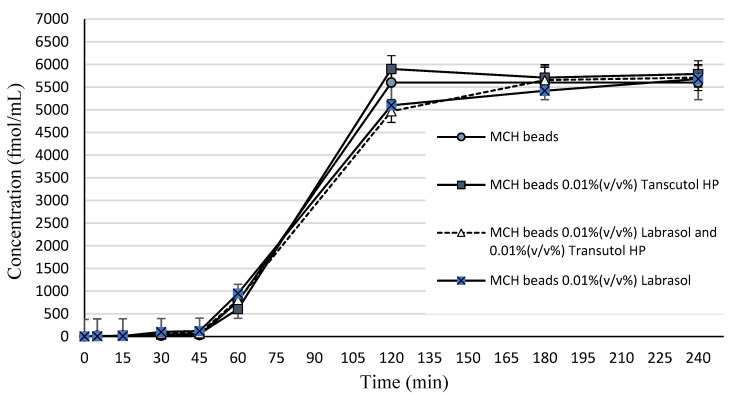
In vitro dissolution profile of MCH from sodium-alginate beads in phosphate buffer solution (pH = 6.80). Significant peptide release started after 60 min and reached a plateau after 120 min. Each data point represents the mean ± SD, *n* = 3.

In vitro peptide release experiment was conducted in buffer phosphate solution (pH = 6.80). Samples were tested by RIA. Figure 5 represents the drug concentration released from alginate beads by time. Dissolution profiles were determined by plotting the experimental data. According to the data presented, a biphasic release pattern can be observed. MCH was released in a prolonged manner, since in the first 60 min no significant release could be measured. After one hour, an initial burst release started, where 68.30% of the incorporated MCH was released from the beads. The release ratio of MCH after 120 min was significantly low.

### 3.7. Enzymatic Stability

**Figure 6:**
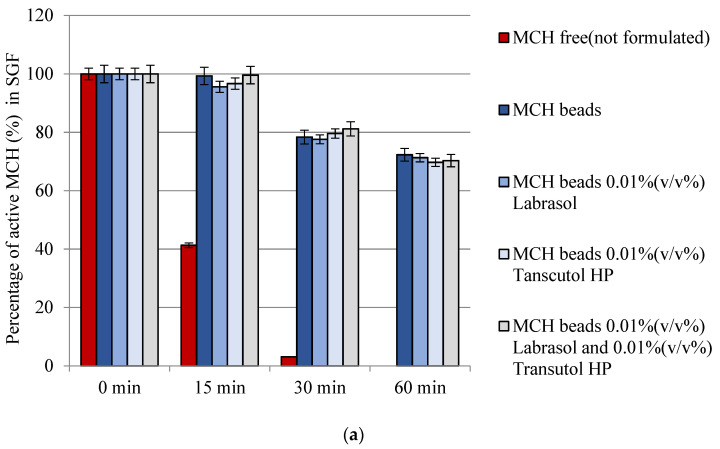

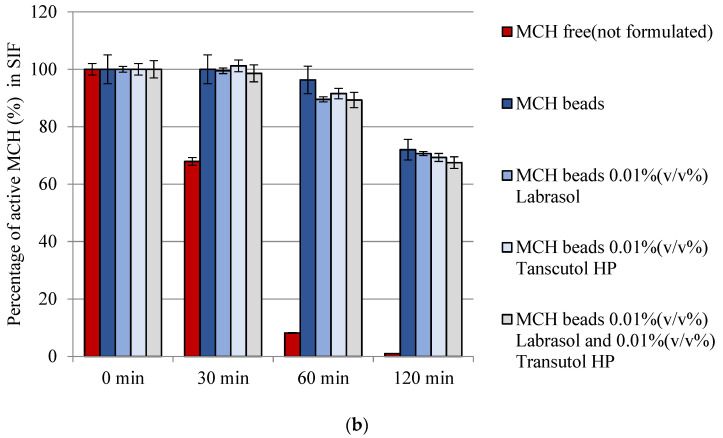
Enzymatic stability of melanin concentrating hormone (MCH) encapsulated in sodium-alginate beads with free (not formulated) MCH as a control: (**a**) in SGF medium; (**b**) in SIF medium. Each data point represents the mean ± SD, *n* = 3.

It had been shown that only 3.00 ± 0.50% and 8.00 ± 0.50% active MCH could be measured after 30 min incubation in SGF and SIF, respectively, form the free (not formulated) MCH samples. Peptide was completely degraded after 1 h incubation in SGF, while only 1.00 ± 0.50% MCH recovery occurred after 2 h incubation with SIF. Our measurement demonstrated that bead formulations are able to protect the model peptide. In case of each formulation at least 70% of MCH was protected from SGF and SIF degradation. Figure 6 represents the results of the study.

### 3.8. MTT Viability Assay

**Figure 7:**
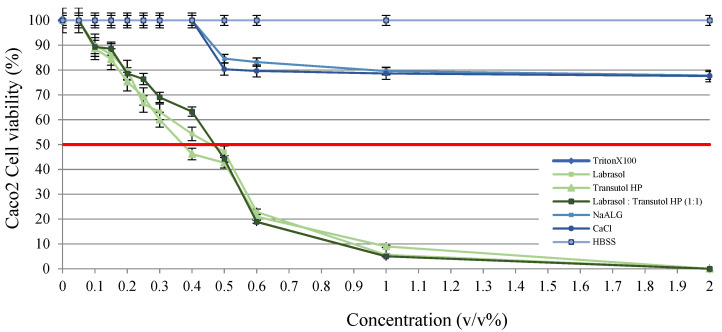
Results of in vitro cytotoxicity measurement of the applied excipients and bead components for the formulation of sodium-alginate beads containing MCH. Bead forming sodium-alginate and calcium chloride dehydrate did not show substantial toxicity, while penetration enhancer excipients (Labrasol, Transcutol HP) seemed to be toxic at higher concentration. Triton-X was used as a positive control; the phosphate-buffered solution (PBS)-treated group was the negative control. Each data point represents the mean ± SD, *n* = 10.

**Figure 8:**
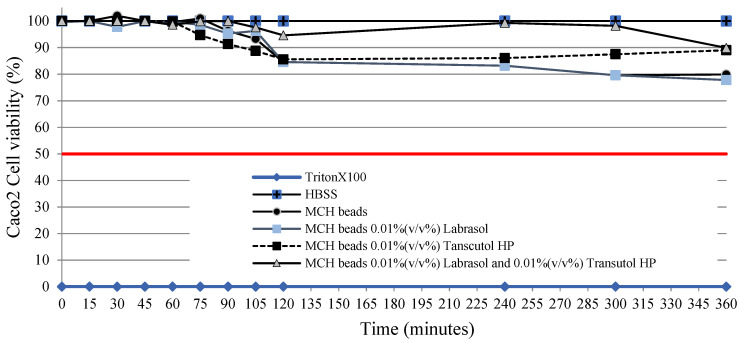
Results of a 6 h treatment of Caco-2 intestinal epithelial cells with MCH beads, MCH beads containing 0.01% (*v*/*v*%) Labrasol, MCH beads containing 0.01% (*v*/*v*%) Transcutol HP, and MCH beads containing 0.01% (*v*/*v*%) Labrasol and 0.01% (*v*/*v*%) Transcutol HP.

According to the performed cell viability measurements it was demonstrated that all of the selected excipients are safe in the applied concentration. Although concentration-dependent toxicity can be observed, sodium-alginate and calcium-chloride dihydrate did not show relevant toxicity, not even at higher concentrations. Figure 7 demonstrates that penetration enhancers showed obvious toxicity at higher concentrations; thus, IC50 values were evaluated. IC50 values of the excipients were determined as Labrasol 0.41 (*v*/*v*%), Transcutol HP 0.37 (*v*/*v*%) and 1:1 ratio blended Labrasol:Transcutol HP composition 0.35 (*v*/*v*%) in our experiments. According to Figure 8, the formulated drug delivery systems with 0.01 (*v*/*v*%) of penetration enhancer content did not resulted in disadvantageous changes to the monolayer.

### 3.9. Permeability Test

**Figure 9:**
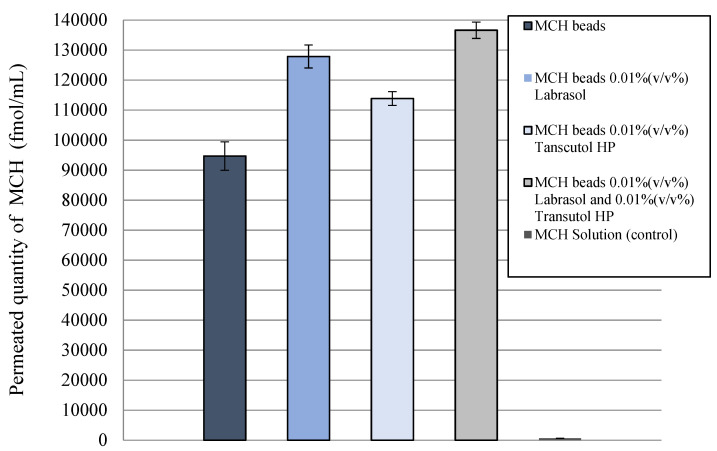
Evaluation of permeability of MCH across Caco-2 epithelial cell layers treated with the different formulations for 1 h. Increased peptide permeability was observed when penetration enhancers were added to the compositions. Each data point represents the mean ± SD, *n* = 10.

Before the permeability test, Caco-2 monolayers on the inserts showed high (600 Ω×cm^2^) TEER values, indicating tight barrier properties. Differences in the penetrated drug concentration is represented in Figure 9. The permeability of MCH encapsulated in alginate beads was significantly higher than that for MCH solution. An increased MCH permeability was measured in the presence of the penetration enhancer, suggesting the opening of a paracellular pathway.

### 3.10. Transepithelial Electrical Resistance Measurements

**Figure 10:**
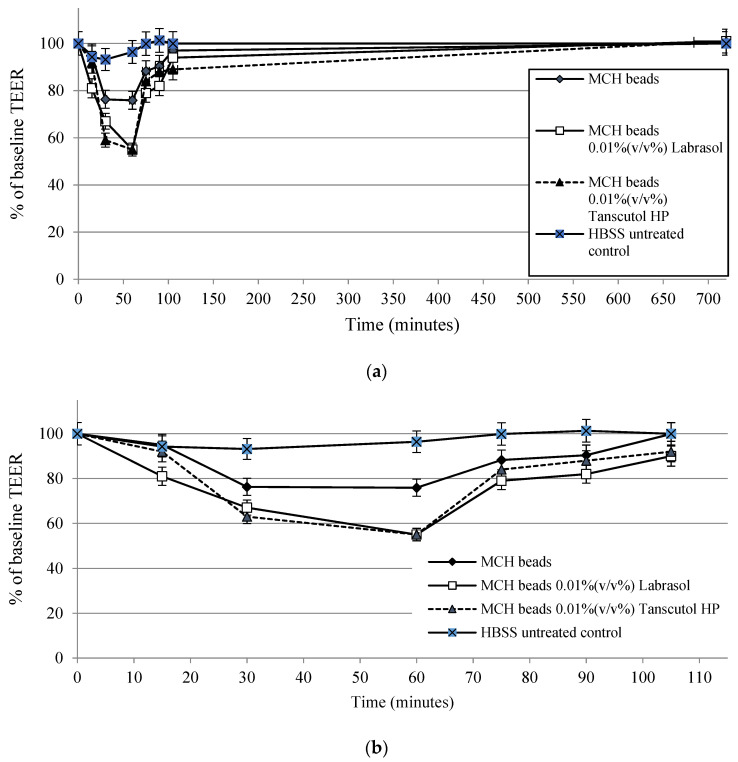
Transepithelial electrical resistance of Caco-2 intestinal epithelial cells: (**a**) regarding the whole experiment; (**b**) focusing on the first 100 min. Each data point represents the mean ± SD, *n* = 5.

The membrane integrity of adenocarcinoma cells was measured using TEER measurements. After 30 min, compositions caused a significant decrease of transepithelial electrical resistance. The follow-up measurements demonstrated that TEER values increased in fresh DMEM after 60 min. TEER values of MCH beads-treated monolayers increased immediately after the treatment. Moreover, transepithelial electric resistance values increased above 90% of the baseline at the end of experiment. Results of TEER measurements are presented in Figure 10.

## 4. Discussion

The encapsulation of peptides into bioadhesive polymer carrier systems is a preferable and widespread way to improve the oral bioavailability of peptide drugs due to their beneficial properties [42]. The objective of the development was to construct suitable carrier systems according to the abovementioned aspects. As model Melanin concentrating hormone (MCH) peptide was selected. MCH-loaded alginate beads were prepared using a controlled polymerization method with a Büchi Encapsulator B-395 Pro apparatus. Our measurements stated that the value of the equilibrium water uptake depends on the particle size of beads. Due to the applicability, a 200 µm diameter was found to be ideal. Performed SEM and DLS measurements [43] confirmed that the morphology is appropriate due to the expectations and the particle size of the formulated microbeads being close to the theoretical 200 µm. Performed measurements demonstrated that the applied alginate-bead formulation method resulted in at least 56% encapsulation efficiency of MCH. Blended penetration enhancers did not affect the closing ability. We can state that our formulations are stable environments to encapsulate peptide drugs. Moreover, these compositions could successfully shelter the peptide from the harsh environment of the simulated conditions of the gastrointestinal tract according to our enzyme resistance tests. Peptide release tests demonstrated that during the first hour, 68.30% of incorporated MCH was disengaged. Due to biosafety having a high priority in the case of novel pharmaceutical formulations, the toxicity of the selected excipients and compositions was evaluated by various in vitro methods [44]. Our results confirmed that the formulated compositions are safe due to the fact that the compositions did not cause disadvantageous changes to the Caco-2 layers. Moreover, during our transmembrane permeability tests, it was demonstrated that the permeation of alginate-encapsulated MCH was significantly higher than native MCH solution. Moreover, an increased MCH permeability was measured in the presence of a penetration enhancer, suggesting the opening of paracellular pathway [45]. This phenomenon was confirmed by our previous immunohistochemistry results as well [46]. To exclude the possibility of membrane damage in the background of this result, transepithelial electrical resistance follow-up measurements were performed. These results indicate that the barrier properties of the monolayers completely recovered within 12 h after penetration enhancer treatment. Our transepithelial electrical resistance measurements proved that the permeation enhancement effect of Labrasol and Transcutol HP did not lead to an irreversible disruption of the Caco-2 monolayers. These results suggest that the alginate beads are promising tools to protect peptide derivatives against GI degradation. Punctiliously selected excipients are able to ensure the biosafety and the appropriate physical properties. Prudent selection of penetration enhancers might lead to improved absorption by reversible alteration of barrier functions. These formulations can be vital tools for novel peptide delivery systems with excellent pharmaceutical properties. Moreover, they are able to improve patient’s adherence and experience.

## 5. Conclusions

In our present study, we designed innovative solid oral delivery systems for peptide delivery with improved oral bioavailability. According to the results, we managed to show that alginate beads are able to protect melanin concentrating hormones during the formulation. Moreover, the peptide was protected against gastric and intestinal enzymatic degradation as well. The combination of alginate carriers with amphiphilic surfactants has not been described yet. Our results demonstrated that these penetration enhancers might play a key role in bioavailability improvement. This result might ensure useful data for further formulation as well. The aims of our following experiments are to improve the encapsulation efficiency by excluding or changing those formulation steps that lead to MCH loss. Furthermore, possible methods of particle size reduction and other penetration enhancer combinations will be tested to improve efficiency.

## Figures and Tables

**Table 1:** Composition of the selected blends.

Selected Blends	Sodium-Alginate Solution	Labrasol	Transcutol HP
MCH beads	40 mL	-	-
MCH beads + 0.01% (*v*/*v*%) Labrasol	40 mL	0.01% (*v*/*v*%)	-
MCH beads + 0.01% (*v*/*v*%) Transcutol HP	40 mL	-	0.01% (*v*/*v*%)
MCH beads + 0.01% (*v*/*v*%) Labrasol and 0.01% (*v*/*v*%) Transcutol HP	40 mL	0.01% (*v*/*v*%)	0.01% (*v*/*v*%)

**Table 2:** Results of laser diffraction particle size distribution.

Sample	d(0.1) µm	d(0.9) µm	d(4.3) µm
MCH beads	144.03 ± 7.37	193.32 ± 8.25	209.65 ± 28
MCH beads + 0.01% (*v*/*v*%) Labrasol	146.09 ± 6.21	198.42 ± 3.63	208.23 ± 1.56
MCH beads + 0.01% (*v*/*v*%) Transcutol HP	141.52 ± 3.97	205.26 ± 2.83	220.30 ± 3.85
MCH beads + 0.01% (*v*/*v*%) Labrasol and 0.01% (*v*/*v*%) Transcutol HP	146.71 ± 7.02	199.47 ± 4.23	211.10 ± 1.19

## References

[1] Deb P.K., Al-Attraqchi O., Chandrasekaran B., Paradkar A., Tekade R.K. (2019). Protein/Peptide Drug Delivery Systems. Basic Fundamentals of Drug Delivery.

[2] Brayden D.J., Mrsny R.J. (2011). Oral peptide delivery: Prioritizing the leading technologies. Ther. Deliv..

[3] Shaji J., Patole V. (2008). Protein and peptide drug delivery: Oral approaches. Indian J. Pharm. Sci..

[4] Mahato R.I., Narang A.S., Thoma L., Miller D.D. (2003). Emerging Trends in Oral Delivery of Peptide and Protein Drugs. Crit. Rev. Ther. Drug Carrier Syst..

[5] Aungst B.J., Saitoh H., Burcham D.L., Huang S.-M., Mousa S.A., Hussain M.A. (1996). Enhancement of the intestinal absorption of peptides and nonpeptides. J. Control. Release.

[6] Bruno B.J., Miller G.D., Lim C.S. (2013). Basics and recent advances in peptide and protein drug delivery. Ther. Deliv..

[7] Brayden D.J., Hill T.A., Fairlie D.P., Maher S., Mrsny R.J. (2020). Systemic delivery of peptides by the oral route: Formulation and medicinal chemistry approaches. Adv. Drug Deliv. Rev..

[8] Anderson S.L., Beutel T.R., Trujillo J.M. (2020). Oral semaglutide in type 2 diabetes. J. Diabetes Complicat..

[9] Ways T.M., Lau W., Khutoryanskiy V. (2018). Chitosan and Its Derivatives for Application in Mucoadhesive Drug Delivery Systems. Polymers.

[10] Greimel A., Werle M., Bernkop-Schnürch A. (2010). Oral peptide delivery: In-vitro evaluation of thiolated alginate/poly (acrylic acid) microparticles. J. Pharm. Pharmacol..

[11] Coppi G., Iannuccelli V., Leo E., Bernabei M.T., Cameroni R. (2001). Chitosan-Alginate Microparticles as a Protein Carrier. Drug Dev. Ind. Pharm..

[12] Banerjee A., Lee J., Mitragotri S. (2016). Intestinal mucoadhesive devices for oral delivery of insulin. Bioeng. Transl. Med..

[13] Park C.G., Huh B.K., Kim S.-N., Lee S.H., Hong H.R., Choy Y. (2017). Bin Nanostructured mucoadhesive microparticles to enhance oral drug bioavailability. J. Ind. Eng. Chem..

[14] Arnaldi P., Pastorino L., Monticelli O. (2020). On an effective approach to improve the properties and the drug release of chitosan-based microparticles. Int. J. Biol. Macromol..

[15] Gupta V., Hwang B.-H., Doshi N., Banerjee A., Anselmo A.C., Mitragotri S. (2016). Delivery of Exenatide and Insulin Using Mucoadhesive Intestinal Devices. Ann. Biomed. Eng..

[16] Yu L., Sun Q., Hui Y., Seth A., Petrovsky N., Zhao C.-X. (2019). Microfluidic formation of core-shell alginate microparticles for protein encapsulation and controlled release. J. Colloid Interface Sci..

[17] Zhang Y., Wei W., Lv P., Wang L., Ma G. (2011). Preparation and evaluation of alginate–chitosan microspheres for oral delivery of insulin. Eur. J. Pharm. Biopharm..

[18] Gombotz W. (1998). Protein release from alginate matrices. Adv. Drug Deliv. Rev..

[19] McClements D.J. (2018). Encapsulation, protection, and delivery of bioactive proteins and peptides using nanoparticle and microparticle systems: A review. Adv. Colloid Interface Sci..

[20] Rahmani V., Sheardown H. (2018). Protein-alginate complexes as pH-/ion-sensitive carriers of proteins. Int. J. Pharm..

[21] Somo S.I., Langert K., Yang C.-Y., Vaicik M.K., Ibarra V., Appel A.A., Akar B., Cheng M.-H., Brey E.M. (2018). Synthesis and evaluation of dual crosslinked alginate microbeads. Acta Biomater..

[22] Moya M.L., Morley M., Khanna O., Opara E.C., Brey E.M. (2012). Stability of alginate microbead properties In Vitro. J. Mater. Sci. Mater. Med..

[23] Fenyvesi Z., Auner A., Schmalz D., Pásztor E., Csóka G., Gyires K., Marton S., Klebovich I., Antal I. (2009). Preparation of pH-sensitive beads for NSAID using three-component gel systems. J. Pharm. Sci..

[24] Agarwal V., Khan M.A. (2001). Current Status of the Oral Delivery of Insulin. Pharm. Technol..

[25] Ibrahim Y.H.-E.Y., Regdon G., Hamedelniel E.I., Sovány T. (2020). Review of recently used techniques and materials to improve the efficiency of orally administered proteins/peptides. DARU J. Pharm. Sci..

[26] Han Y., Gao Z., Chen L., Kang L., Huang W., Jin M., Wang Q., Bae Y.H. (2019). Multifunctional oral delivery systems for enhanced bioavailability of therapeutic peptides/proteins. Acta Pharm. Sin. B.

[27] Christophersen P.C., Zhang L., Müllertz A., Nielsen H.M., Yang M., Mu H. (2014). Solid Lipid Particles for Oral Delivery of Peptide and Protein Drugs II—The Digestion of Trilaurin Protects Desmopressin from Proteolytic Degradation. Pharm. Res..

[28] Casatti C., Elias C., Sita L., Frigo L., Furlani V.C., Bauer J., Bittencourt J. (2002). Distribution of melanin-concentrating hormone neurons projecting to the medial mammillary nucleus. Neuroscience.

[29] Marsh D.J., Weingarth D.T., Novi D.E., Chen H.Y., Trumbauer M.E., Chen A.S., Guan X.-M., Jiang M.M., Feng Y., Camacho R.E. (2002). Melanin-concentrating hormone 1 receptor-deficient mice are lean, hyperactive, and hyperphagic and have altered metabolism. Proc. Natl. Acad. Sci. USA.

[30] Ludwig D.S., Tritos N.A., Mastaitis J.W., Kulkarni R., Kokkotou E., Elmquist J., Lowell B., Flier J.S., Maratos-Flier E. (2001). Melanin-concentrating hormone overexpression in transgenic mice leads to obesity and insulin resistance. J. Clin. Investig..

[31] Tritos N.A., Maratos-Flier E. (1999). Two important systems in energy homeostasis: Melanocortins and melanin-concentrating hormone. Neuropeptides.

[32] Bradley R.L., Mansfield J.P.R., Maratos-Flier E., Cheatham B. (2002). Melanin-concentrating hormone activates signaling pathways in 3T3-L1 adipocytes. Am. J. Physiol. Metab..

[33] Jiang H., Gallet S., Klemm P., Scholl P., Folz-Donahue K., Altmüller J., Alber J., Heilinger C., Kukat C., Loyens A. (2020). MCH Neurons Regulate Permeability of the Median Eminence Barrier. Neuron.

[34] De Lartigue G. (2014). Putative roles of neuropeptides in vagal afferent signaling. Physiol. Behav..

[35] Lelesz B., Szilvássy Z., Tóth G.K., Tóth A., Enyedi A., Felszeghy E., Varga A., Juhász B., Németh J. (2016). Radioanalytical methods for the measurement of melanin concentrating hormone (MCH) and detection its receptor in rat tissues. J. Radioanal. Nucl. Chem..

[36] Kozlowska J., Prus W., Stachowiak N. (2019). Microparticles based on natural and synthetic polymers for cosmetic applications. Int. J. Biol. Macromol..

[37] McCartney F., Jannin V., Chevrier S., Boulghobra H., Hristov D.R., Ritter N., Miolane C., Chavant Y., Demarne F., Brayden D.J. (2019). Labrasol^®^ is an efficacious intestinal permeation enhancer across rat intestine: Ex Vivo and In Vivo rat studies. J. Control. Release.

[38] Sullivan D.W., Gad S.C., Julien M. (2014). A review of the nonclinical safety of Transcutol^®^, a highly purified form of diethylene glycol monoethyl ether (DEGEE) used as a pharmaceutical excipient. Food Chem. Toxicol..

[39] Boukamp P., Petrussevska R.T., Breitkreutz D., Hornung J., Markham A., Fusenig N.E. (1988). Normal keratinization in a spontaneously immortalized aneuploid human keratinocyte cell line. J. Cell Biol..

[40] Taqieddin E., Amiji M. (2004). Enzyme immobilization in novel alginate–chitosan core-shell microcapsules. Biomaterials.

[41] Ismail R., Bocsik A., Katona G., Gróf I., Deli M.A., Csóka I. (2019). Encapsulation in Polymeric Nanoparticles Enhances the Enzymatic Stability and the Permeability of the GLP-1 Analog, Liraglutide, Across a Culture Model of Intestinal Permeability. Pharmaceutics.

[42] Reinholz J., Landfester K., Mailänder V. (2018). The challenges of oral drug delivery via nanocarriers. Drug Deliv..

[43] Mammadov R., Tekinay A.B., Dana A., Guler M.O. (2012). Microscopic characterization of peptide nanostructures. Micron.

[44] Riss T.L., Moravec R.A., Niles A.L., Duellman S., Benink H.A., Worzella T.J., Minor L. (2004). Cell Viability Assays. Assay Guidance Manual.

[45] Alvi M.M., Chatterjee P. (2014). A Prospective Analysis of Co-Processed Non-Ionic Surfactants in Enhancing Permeability of a Model Hydrophilic Drug. AAPS PharmSciTech.

[46] Ujhelyi Z., Fenyvesi F., Váradi J., Fehér P., Kiss T., Veszelka S., Deli M., Vecsernyés M., Bácskay I. (2012). Evaluation of cytotoxicity of surfactants used in self-micro emulsifying drug delivery systems and their effects on paracellular transport in Caco-2 cell monolayer. Eur. J. Pharm. Sci..

